# Value of Hybrid Imaging with PET/CT to Guide Percutaneous Revascularization of Chronic Total Coronary Occlusion

**DOI:** 10.1007/s12410-015-9340-2

**Published:** 2015-05-26

**Authors:** Wijnand J. Stuijfzand, Pieter G. Raijmakers, Roel S. Driessen, Niels van Royen, Alexander Nap, Albert C. van Rossum, Paul Knaapen

**Affiliations:** Department of Cardiology, VU University Medical Center, De Boelelaan 1117, 1081 HV Amsterdam, The Netherlands; Radiology & Nuclear Medicine, VU University Medical Center, Amsterdam, The Netherlands

**Keywords:** Coronary artery disease, Invasive coronary angiography, Chronic total occlusion, Percutaneous coronary intervention, Coronary computed tomography angiography, Positron emission tomography

## Abstract

Chronic total coronary occlusions (CTO) are documented in approximately one fifth of diagnostic invasive coronary angiographies (ICA). Percutaneous coronary interventions (PCI) of CTO are challenging and are accompanied by higher complication and lower success rates in comparison with non-CTO PCI. Scrutinous evaluation of ischemia and viability to justify percutaneous revascularization is therefore of importance to select eligible patients for such a procedure. Furthermore, knowledge of the anatomical features of the occlusion may predict the chances of success of PCI CTO and could even guide the procedural strategy to augment the likelihood of recanalization. Positron emission tomography (PET) is unequivocally accepted as the reference standard for ischemia and viability testing, whereas coronary computed tomography angiography (CCTA) currently allows for non-invasive detailed three-dimensional imaging of the coronary anatomy that adds morphological information over two-dimensional ICA. Hybrid PET/CT could therefore be useful for optimal patient selection as well as procedural planning. This review discusses the potential value of PET/CT to guide PCI in CTOs.

## Introduction

Coronary artery disease (CAD) is the leading cause of death in the Western world. In 18–35 % of patients with known or suspected CAD, a chronic total coronary occlusion (CTO) is involved [1–4]. A CTO is defined as native coronary artery with absent or minimal antegrade blood flow for >12-week duration [5, 6]. Patients with CTOs are treated differently from non-occlusive CAD and are less likely to undergo revascularization, either percutaneously or surgically. For CTO patients who are offered revascularization, coronary artery bypass grafting surgery (CABG) is the mainstay with a 3:1 ratio [1]. The reluctance of physicians to refer patients for revascularization is based on the false assumption that symptoms are easily controlled by optimal medical therapy (OMT) and that the myocardium subtended by the CTO artery is often non-viable and/or non-ischemic that would not benefit from revascularization. Owing to the traditionally low success rate of about 50–70 % and relatively high complication rate, percutaneous coronary intervention (PCI) of CTO is empirically attempted in less than 15 % of eligible patients [1, 7–12]. However, successful recanalization of a CTO is accompanied by symptom relief, recovery of left ventricular (LV) function, and improved survival as opposed to patients in whom the procedure was unsuccessful [3, 7, 13–21].

In recent years, advances in guide wire technology and implementation of dissection and re-entry techniques have resulted in augmented success rates exceeding 90 % in the hands of experienced CTO centers [22–24]. Nonetheless, these procedures are still accompanied by slightly higher complication rates, contrast and radiation burden, and costs in comparison with non-CTO PCI [5, 25–27]. Scrutinous evaluation of ischemia and viability to justify percutaneous revascularization is therefore of importance to select eligible patients for such a procedure. Furthermore, knowledge of the anatomical features of the occlusion may predict the chances of success and could even guide the procedural strategy to augment the likelihood of recanalization in an efficient manner [28•, 29••].

The combination of hybrid positron emission tomography (PET) and coronary computed tomography angiography (CCTA) nowadays allows for the accurate detection and quantification of myocardial ischemia and viability in conjunction with coronary anatomy and morphology near simultaneously. As such, hybrid PET/CT could prove useful in the clinical work-up of CTO patients to determine eligibility and plan procedural strategy. This review discusses the potential value of PET/CT to guide PCI CTO.

## Selection of Eligible Patients for Revascularization of CTO

**Fig. 1:**
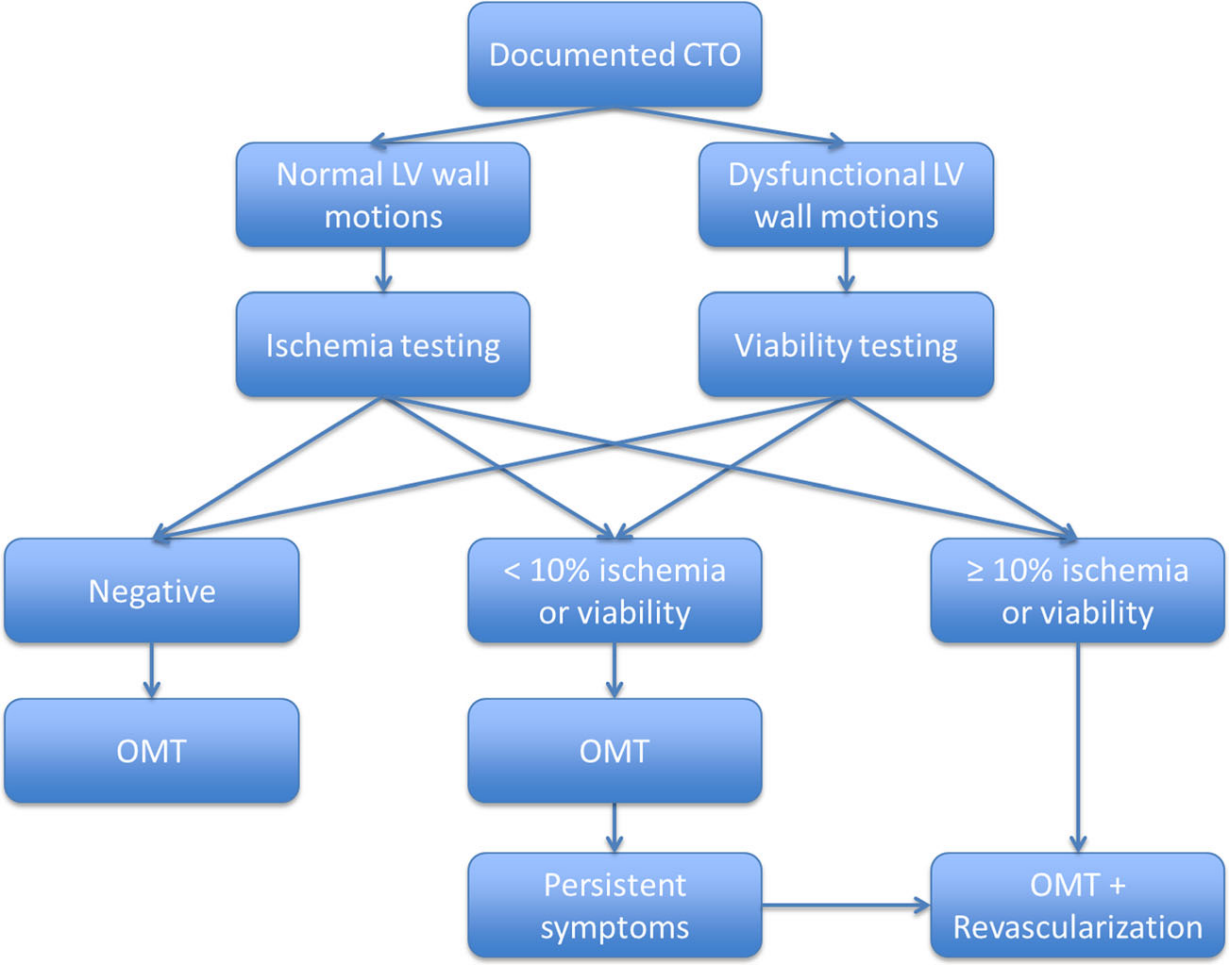
Proposed diagnostic and treatment algorithm in patients with a documented chronic total coronary occlusion (*CTO*). *LV* left ventricular, *OMT* optimal medical therapy

The decision scheme on the diagnostic work-up for CTO patients to evaluate eligibility for revascularization is depicted in Fig. 1. Please note that this algorithm is based on current international guidelines and is not specifically targeted to CTO patients but to obstructive CAD in general. Once the diagnosis of a CTO has been established, usually through a diagnostic invasive coronary angiogram (ICA), information pertaining LV function is mandatory. Normal regional LV function of myocardium subtended by the CTO by definition excludes non-viability. The decision to proceed to a revascularization procedure is then merely based on the presence and extent of ischemia. Without documented ischemia, such a procedure is futile and even hazardous [30] as the patient is subjected to potential procedural complications without a clear benefit and OMT suffices. In the majority of cases, however, ischemia is observed. Even the presence of well-developed collaterals does generally not protect the myocardium against ischemia. An increase in collateral flow reserve during pharmacological stress has been shown to occur in only 7 % of patients, while coronary steal can be documented in a third [31, 32]. When the jeopardized myocardium comprises more than 10 % of the LV, revascularization on top of OMT is considered appropriate irrespective of symptoms [33]. Retrospective analysis of myocardial perfusion studies in large registries have revealed that such an ischemic burden holds prognostic relevance and revascularization is associated with improved outcome [34]. When the extent of ischemia is less pronounced (i.e., < 10 %), an initial treatment strategy of OMT is justified and revascularization only contemplated after inadequate response to medical therapy.

Conversely, when patients exhibit extensive regional wall motion abnormalities in the affected vascular territory, we recommend that the diagnostic work-up focus on myocardial viability rather than ischemia. Without signs of myocardial viability (i.e., previous (near) transmural myocardial infarction), similar to lack of ischemia, revascularization is considered inappropriate as no recovery of function is anticipated [35, 36]. Although, it must be noted that revascularization of non-viable myocardium might halt the process of progressive LV remodelling [37]. The presence of substantial viability warrants revascularization and is accompanied by recovery of LV function and improved outcome as observed in both retrospective and prospective studies [35, 38, 39]. The extent of viability deemed necessary for a clinically relevant improvement in LV ejection fraction (i.e., >5 percentage points) is uncertain, but generally ranges from 10 to 25 % jeopardized myocardium [40, 41]. Small quantities of viability are not associated with recovery of function, but revascularization could be considered to reduce symptoms not controlled by medical therapy.

Although the presented literature supports the utilization of the proposed treatment algorithm, randomized trials on imaging-guided revascularization are scarce. The PET and Recovery Following Revascularization (PARR-2) [42] and a substudy of the Surgical Treatment for Ischemic Heart Failure (STICH) [43] trial have evaluated the impact of viability assessment on clinical outcomes after CABG and were both negative. A nuclear substudy of the Clinical Outcomes Utilizing Revascularization and Aggressive Drug Evaluation (COURAGE) trial suggests that an appreciable reduction of ischemia is accompanied by a favorable outcome after PCI [44]. These results were corroborated for CTO patients who underwent PCI, whereby a baseline ischemic burden >12.5 % in combination with a reduction >5 % yielded the most favorable clinical outcomes [45]. A randomized imaging study on the merits of ischemia-guided revascularization is, however, lacking altogether. The ongoing International Study of Comparative Health Effectiveness with Medical and Invasive Approaches (ISCHEMIA) trial is attempting to establish the value of revascularization in patients with a large ischemic burden in a randomized fashion. More specifically tailored to PCI-CTO, large observational studies have shown that a successful percutaneous recanalization results in improved all-cause mortality, lower MACE rates (relative risk: 0.70), and reduced need for CABG as opposed to unsuccessful attempts [13, 18, 19, 46]. Nonetheless, these studies are all observational in nature and thus prone to confounding. Currently, the Drug-Eluting Stent Implantation Versus Optimal Medical Treatment in Patients with Chronic Total Occlusion (DECISION-CTO) and the Randomized Multicentre Trial to Evaluate the Utilization of Revascularization or Optimal Medical Therapy for the Treatment of Chronic Total Coronary Occlusions (EURO-CTO) trials are the first studies to more accurately assess the surplus clinical value of PCI-CTO over OMT alone in a randomized fashion. The results of the aforementioned studies are eagerly awaited.

## Positron Emission Tomography

Although a large armamentarium of imaging modalities is available to evaluate myocardial perfusion and viability, PET is unequivocally accepted as the reference standard for this task. The following section will place the value of PET within the context of alternative imaging techniques.

### Myocardial Perfusion Imaging

Four tracers in particular have been well validated for myocardial perfusion imaging (MPI) with PET [47•]. Of the available tracers, ^82^Rb, ^13^NH_3_, and H_2_^15^O are the most commonly used. ^18^F-flurpiridaz is an emerging perfusion tracer not yet available for clinical use, but holds great potential and is currently being tested in phase 3 trials [48–50]. Each of these tracers possesses unique characteristics with their individual pros and cons pertaining (costs of) radionuclide production, physical half-life, image quality, radiation exposure, and compatibility with exercise acquisition protocols (Table 1). None of the perfusion tracers excels on all of these features. Choice of tracer is therefore multifactorial and frequently depends on practical and logistical considerations.

**Table 1:** PET tracer characteristics

	H_2_^15^O	^13^NH_3_	^82^Rb	^18^F-flurpiridaz
Half-life	123 s	9.97 min	76 s	110 min
Production	Cyclotron	Cyclotron	Generator	Cyclotron
Kinetics	Freely diffusible, metabolically inert	Metabolically trapped in myocardium	Metabolically trapped in myocardium	Metabolically trapped in myocardium
Mean positron range in tissue	1.1 mm	0.4 mm	2.8 mm	0.2 mm
Scan duration	6 min	20 min	6 min	20 min
Gating/LV function	−	+	+	+
Radiation dose (3D) according to protocol in references	∼0.4 mSv/370 MBq	∼1 mSv/550 MBq	∼0.7 mSv/555 MBq (2D: ∼2.3 mSv/1850 MBq)	∼2.1 mSv/111 MBq (rest) ∼4.6 mSv/244 MBq (stress)
Exercise protocol compatible	−	−	−	+
Quantification	Excellent	Good	Moderate	Very good
Image quality	Good (parametric images)	Very good	Good	Excellent

*PET* positron emission tomography, *H*_*2*_^*15*^ *O* oxygen-15-labeled water, ^*13*^ *NH*_*3*_ 13N-labeled ammonia, ^*82*^ *Rb* ^82^rubidium, *mSv* millisievert, *MBq* megabecquerel

**Fig. 2:**
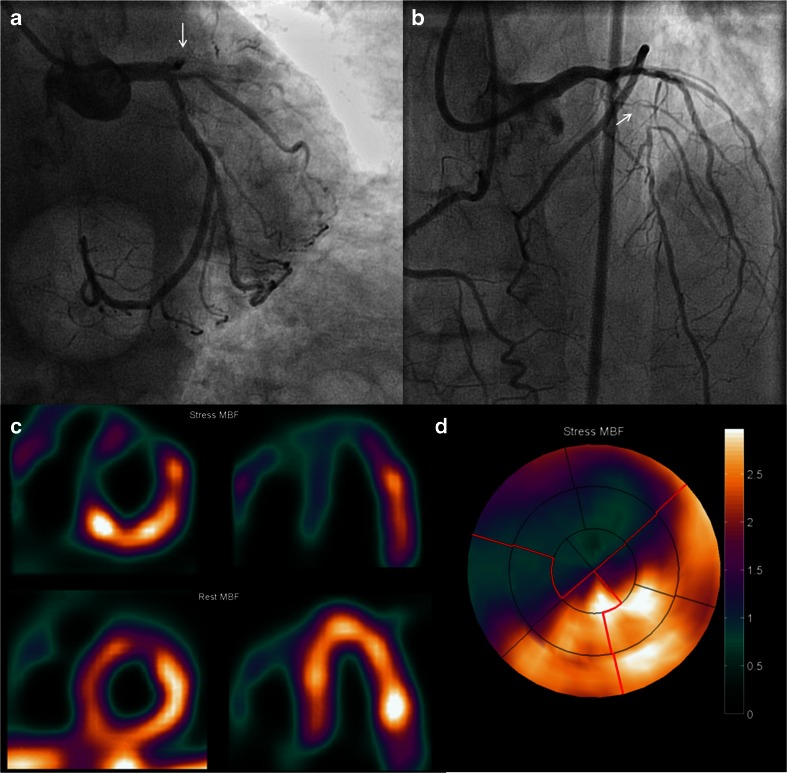
Extensive myocardial ischemia induced by a CTO LAD. Patient with a known CTO LAD for over 7 years with NYHA class II was analyzed with PET to assess the extent of myocardial ischemia before reconsidering (percutaneous) revascularization. **a** The proximal total occlusion of the LAD (*white arrow*). To appreciate collateralization, length, and course (*white arrow*) of the occlusion, a bilateral injection was performed during a diagnostic invasive coronary angiogram (**b**). PET perfusion shows the extensive (**c** and **d**) perfusion defect in presence of a proximal occlusion of the LAD. *CTO* chronic total occlusion, *LAD* left anterior descending artery, *NYHA* New York Heart Association, *PET* positron emission tomography

**Fig. 3:**
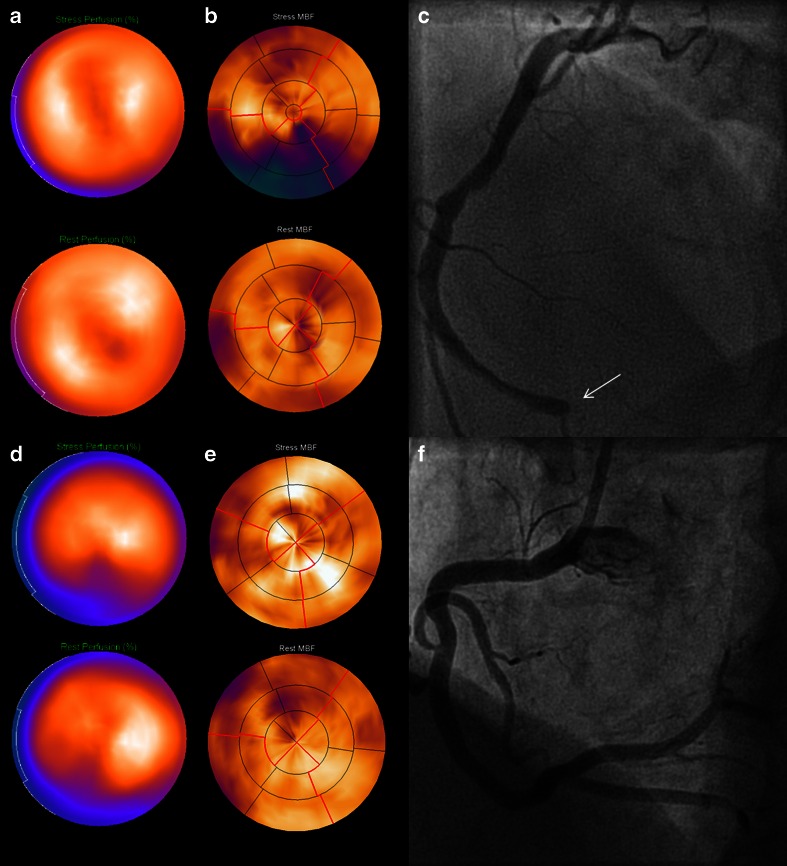
Representative cases of a false-negative and false-positive SPECT result due to limited tracer extraction or attenuation, respectively. False-negative SPECT result (**a**) in case of an unmistakable perfusion defect of the inferior wall on PET (**b**) of a patient with a chronic total occlusion (*white arrow*) of the RCA on ICA (**c**). False-positive SPECT result (**d**) (perfusion defect of the inferior wall) of a patient with normal perfusion on PET (**e**) and no obstructive coronary artery disease on ICA (**f**). *SPECT* single photon emission computed tomography, *RCA* right coronary artery, *ICA* invasive coronary angiography; other abbreviations as in Fig. 2

**Fig. 4:**
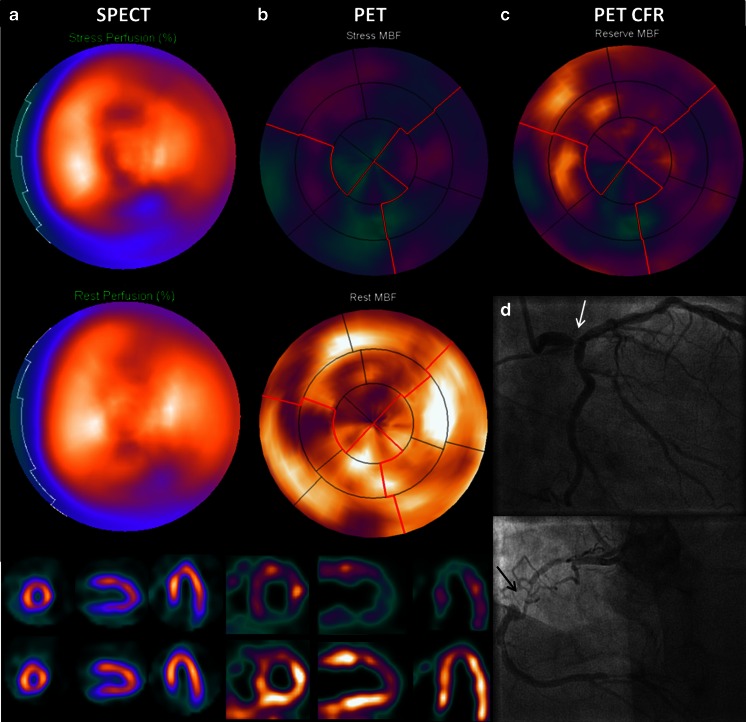
Balanced ischemia on SPECT. Example of a (nearly) normal SPECT (**a**) and severely reduced perfusion on positron emission tomography (PET) (**b**, **c**) in case of a significant left main stenosis (*white arrow*) and a chronic total occlusion of the RCA (*black arrow*) on invasive coronary angiography (**d**). SPECT is hampered by the relative nature of the images, whereas quantification with PET allows for detection of diffusely balanced ischemia. Abbreviations as in Figs. 2 and 3

**Fig. 5:**
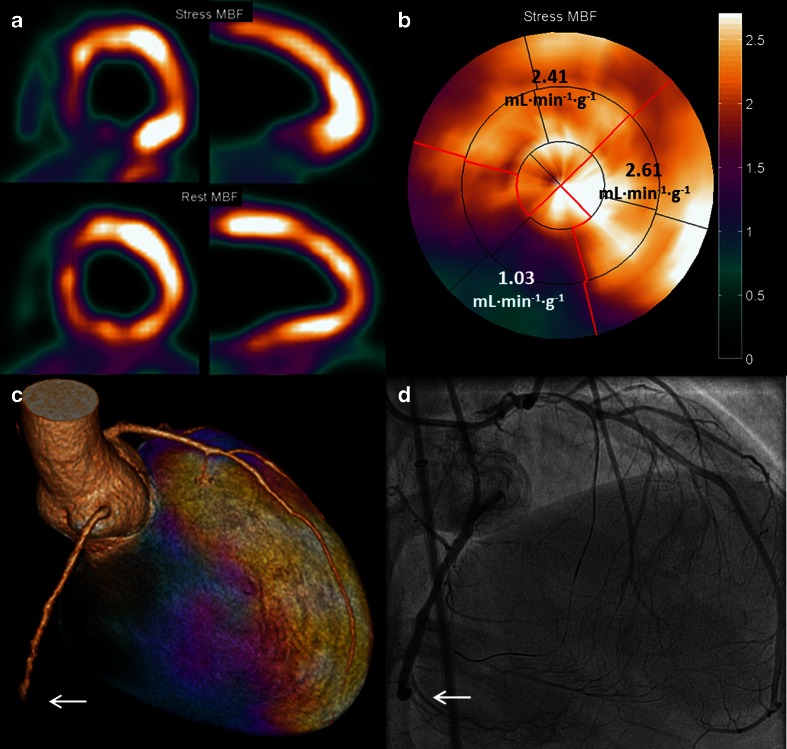
State-of-the-art illustration of fused CCTA and PET of a CTO RCA. Relative and absolute (normal value >2.3 mL min^−1^ g^−1^ [58]) perfusion defect on H_2_^15^O PET perfusion (**a**, **b**) fused with CCTA (**c**) shows the perfusion defect of the left ventricle in relation to the occluded segment of the RCA (*white arrow*). ICA confirmed the precise location of the coronary occlusion (*white arrow*) with bilateral contrast injection (**d**). *CCTA* coronary computed tomography angiography; other abbreviations as in Figs. 2 and 3

Figure 2 shows an example of perfusion PET in a patient with a CTO of the LAD with collateral filling through a right ventricular branch. A large perfusion defect can be observed during hyperemia, whereas resting perfusion is normal. These images justify an attempt to recanalize the artery for prognostic benefit as already alluded to. In the previous section, diagnostic accuracy for MPI with PET to diagnose obstructive CAD has proven to be excellent. Pooled analysis of diagnostic studies displays weighted sensitivity, specificity, NPV, and PPV of 91, 86, 81, and 93 %, respectively [47•]. Moreover, diagnostic performance of PET is superior to the more commonly utilized single photon emission computed tomography (SPECT) MPI. Increased tracer extraction and higher spatial resolution of PET allow detection of subtle perfusion defects that may go undetected with SPECT. In addition, routine attenuation correction of PET significantly reduces false-positive results that are frequently observed with SPECT [51]. Figure 3 shows examples of these phenomena. In recent years, cardiovascular magnetic resonance imaging (CMR) has emerged as an alternative imaging tool for MPI. CMR has the advantage of superior spatial resolution, and perfusion can be visually assessed by first pass imaging of gadolinium-based contrast agents. Nonetheless, a meta-analysis by Jaarsma et al. has demonstrated that PET is the imaging modality of choice to evaluate the functional consequences of CAD with higher performance over SPECT and CMR [52•]. Another distinct advantage of PET is the fact that, next to visual assessment of regional perfusion defects, myocardial blood flow is routine quantified in absolute terms (i.e., in units of mL min^−1^ g^−1^) and allows to calculate coronary flow reserve (CFR) [53–57]. Quantification of perfusion unmasks conditions of balanced ischemia whereby visual interpretation of perfusion images (regardless of the utilized technique like PET, SPECT, or CMR) can be completely normal and yield false-negative results in multi-vessel disease. As patients with CTO frequently exhibit multi-vessel CAD, routine quantification of perfusion with PET aids in the interpretation of a seemingly normal scan (Fig. 4). Furthermore, PET/CT allows for near simultaneous cardiac PET and CCTA to assess perfusion defects in relation to the coronary anatomy (Fig. 5).

### Myocardial Viability Imaging

**Fig. 6:**
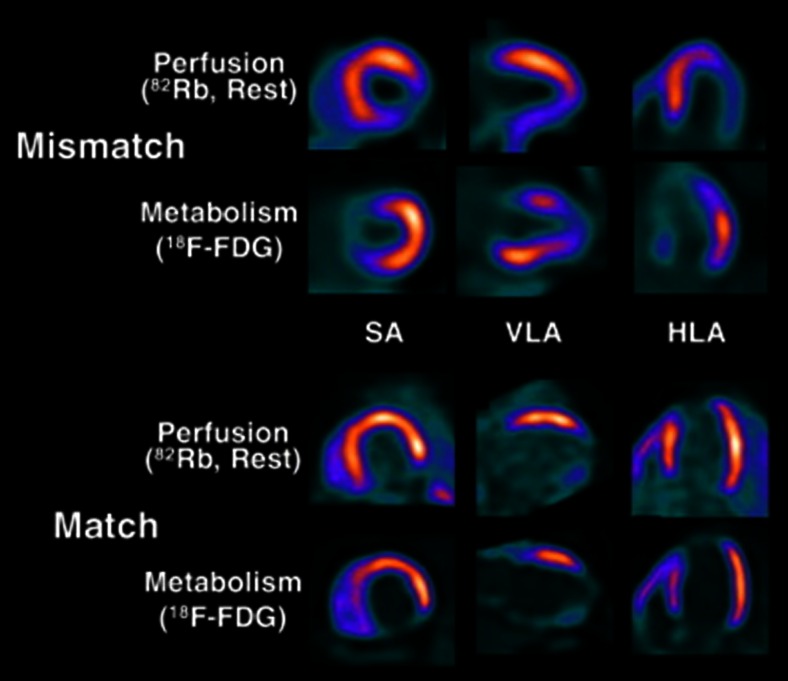
Myocardial viability imaging with PET. Illustration showing a mismatch of resting MBF and glucose metabolism of the inferolateral wall indicating viable myocardium (*top*). A matched resting perfusion and glucose metabolism defect of the inferior wall, indicating non-viable myocardium is shown in the bottom image. ^*82*^ *Rb* ^82^rubidium, ^*18*^ *F-FDG* ^18^F-deoxyglucose, *SA* short axis, *HLA* horizontal long axis, *VLA* vertical long axis; reprint with permission [63]

**Fig. 7:**
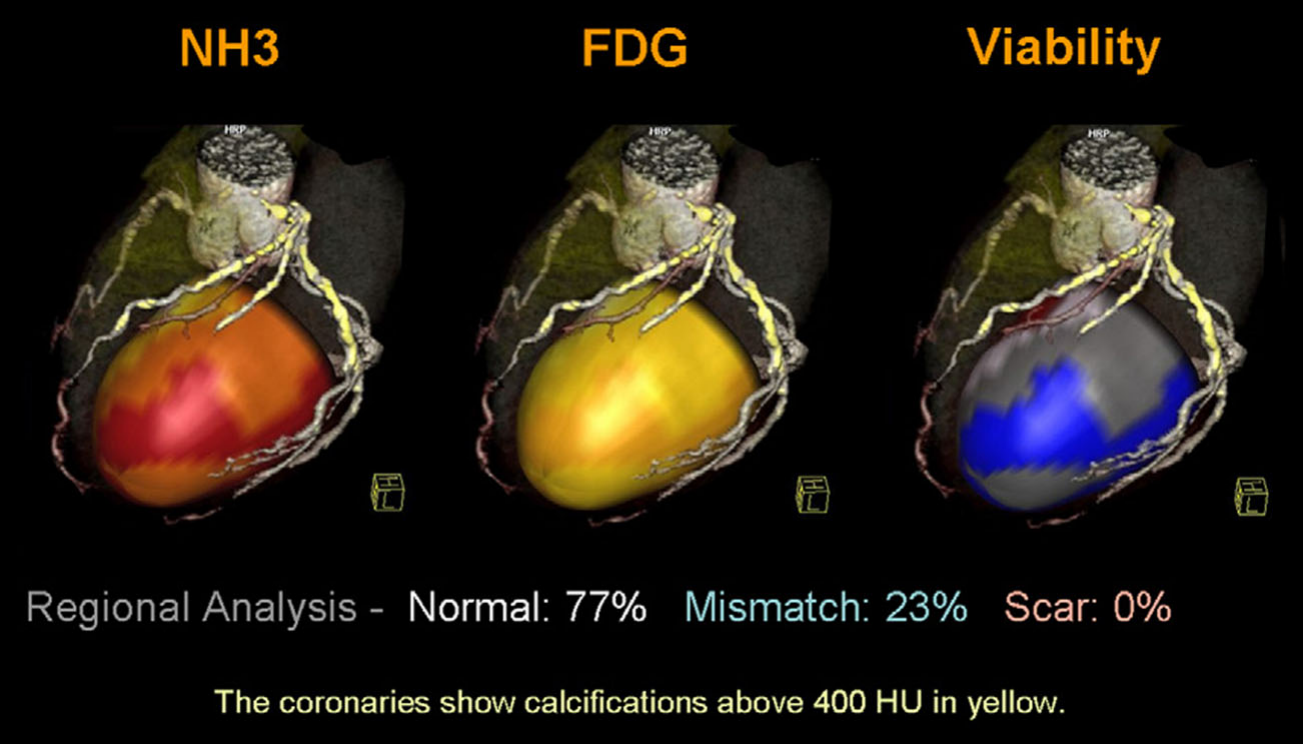
Viability assessment with combined PET perfusion and metabolism. Illustration of simultaneous mapping of coronary anatomy, myocardial perfusion, and metabolism. Due to fused images, it becomes possible to precisely distinguish ischemic viable form non-viable myocardium. ^*13*^ *NH*_*3*_ 13N-labeled ammonia, *HU* Hounsfield units; other abbreviations as in Fig. 6; reprint with permission [64]

In approximately half of CTO patients, wall motion abnormalities are observed with electrocardiographic evidence of myocardial infarction in a third of patients [1]. It is of interest to note that the presence or absence of collaterals does not signify or excludes viability, respectively [59–61]. In such conditions, viability imaging is advocated. Normal resting perfusion basically indicates intact capillary and sarcolemmal membranes and is thus confirmatory of viable myocardium. An irreversible (“fixed”) perfusion defect, however, does not mean that the myocardium is not viable as it may represent either myocardial scarring (non-viable myocardium) or hibernating myocardium, a condition characterized by resting hypoperfusion with preserved or even augmented metabolism. The distinction between these two conditions can be made by metabolic imaging. The most experience for myocardial substrate metabolism has been obtained with the glucose analogue ^18^F-deoxyglucose (^18^F-FDG) [62]. Figure 6 shows the imaging patterns of resting MBF and glucose metabolism in a patient with (mismatch) and without myocardial viability (match). Although nowadays, there are many alternatives for myocardial viability testing (e.g., ^99m^Technetium SPECT, low-dose dobutamine echocardiography, and delayed contrast enhanced CMR), the combination of cardiac perfusion/metabolism PET is still considered the gold standard with the highest diagnostic accuracy. PET/CT offers the additional advantage to fuse the metabolic with the anatomical images to more comprehensively evaluate regional viability in relation to the coronary tree (Fig. 7).

## Coronary Computed Tomography Angiography

**Fig. 8:**
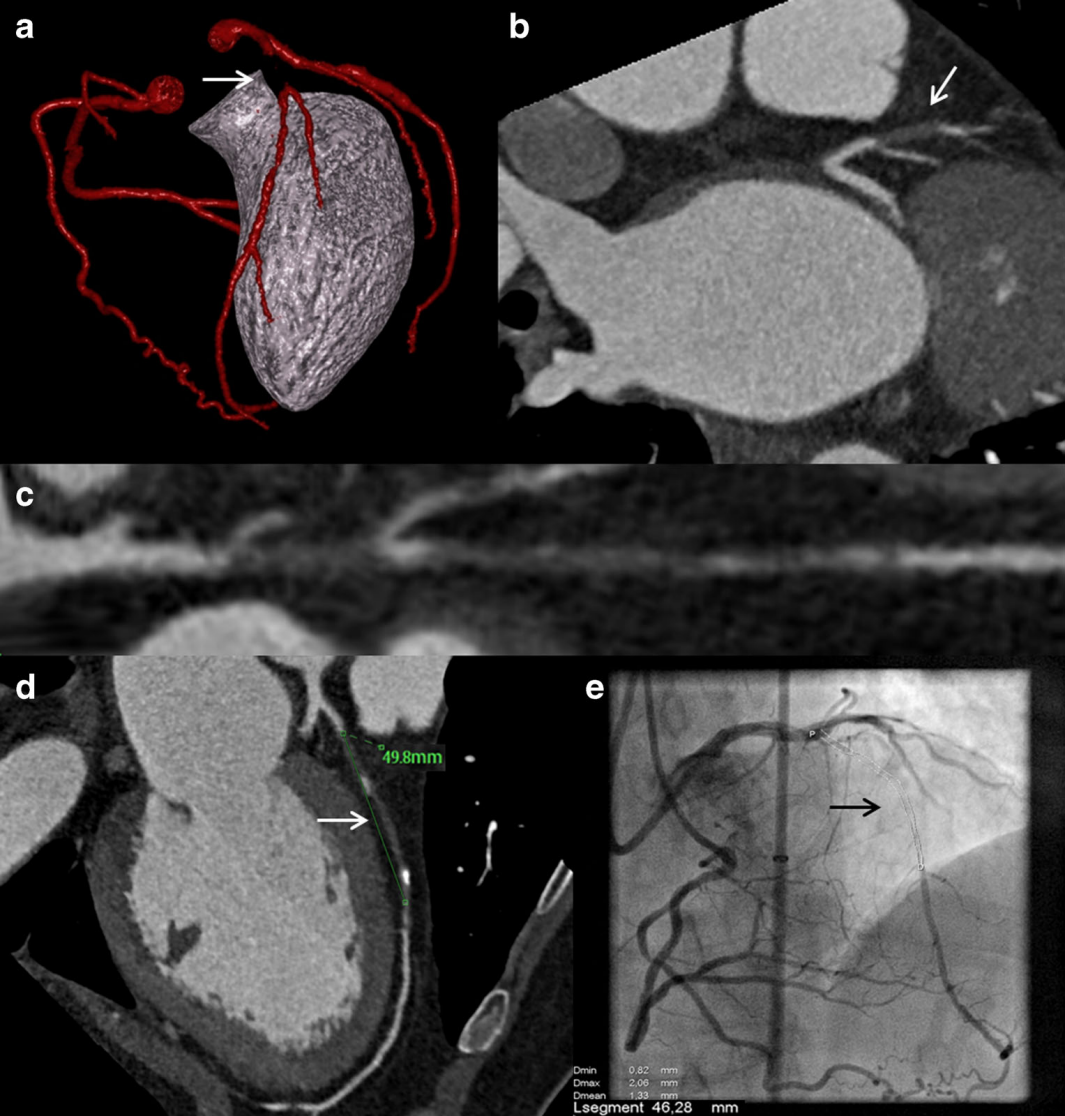
Example of precise occlusion length estimation with CCTA. Three-dimensional reconstruction (**a**), maximum intensity projections (**b**), center line extraction (**c**), and multiplanar reconstructions (**d**) of CCTA accurately assesses coronary course, epicardial collaterals, plaque morphology, and occlusion length in case of a long LAD occlusion (*white arrows*). The occlusion length (*black arrow*) was confirmed with bilateral contrast injection during ICA (**e**). Abbreviations as in Figs. 2, 3, and 5

Over the last decade, CCTA has developed as a valuable non-invasive alternative for the visualization of coronary anatomy. Current multislice CT scanners in combination with modern acquisition protocols enable robust and reproducible assessment of coronary artery morphology with relatively high temporal and spatial resolution accomplished at an acceptable radiation dose [65–68]. CCTA allows to visualize the characteristics of a CTO, which is useful not only for diagnostic purposes but also for planning a potential interventional strategy. CCTA even has some advantages over invasive angiography with three-dimensional reconstructions of the affected segment to fully appreciate the course and length of the occlusion (Fig. 8).

## Definition of CTO on CCTA

A CTO on CCTA is diagnosed if there is complete discontinuity of contrast opacification of the coronary artery lumen in the cross-sectional views, multiplanar reconstructions (MPR), and maximum intensity projections (MIP). The time lapse between the intravenous contrast injection and the contrast bolus tracking setoff results in less dense and reverse contrast opacification distal to the occlusion due to collateral flow [69, 70]. Suspicion of a more recent occluded vessel should rise if there is no distal contrast opacification [71, 72]. Von Erffa et al. showed that an occlusion length of ≥9 mm on CCTA predicted a total occlusion on ICA with 100 % specificity in a group of 40 consecutive patients with a lesion demonstrating complete contrast discontinuity on CCTA. Figure 8 shows the accuracy of occlusion length on CCTA in comparison with ICA. Nevertheless, differentiation between high-grade stenosis and total occlusion remains unreliable due to the relatively limited spatial resolution of CCTA [73], while the presence of true lumen antegrade flow is of clinically importance since it significantly influences the success rate of PCI.

## Angiographic Predictors of Success of PCI CTO

**Fig. 9:**
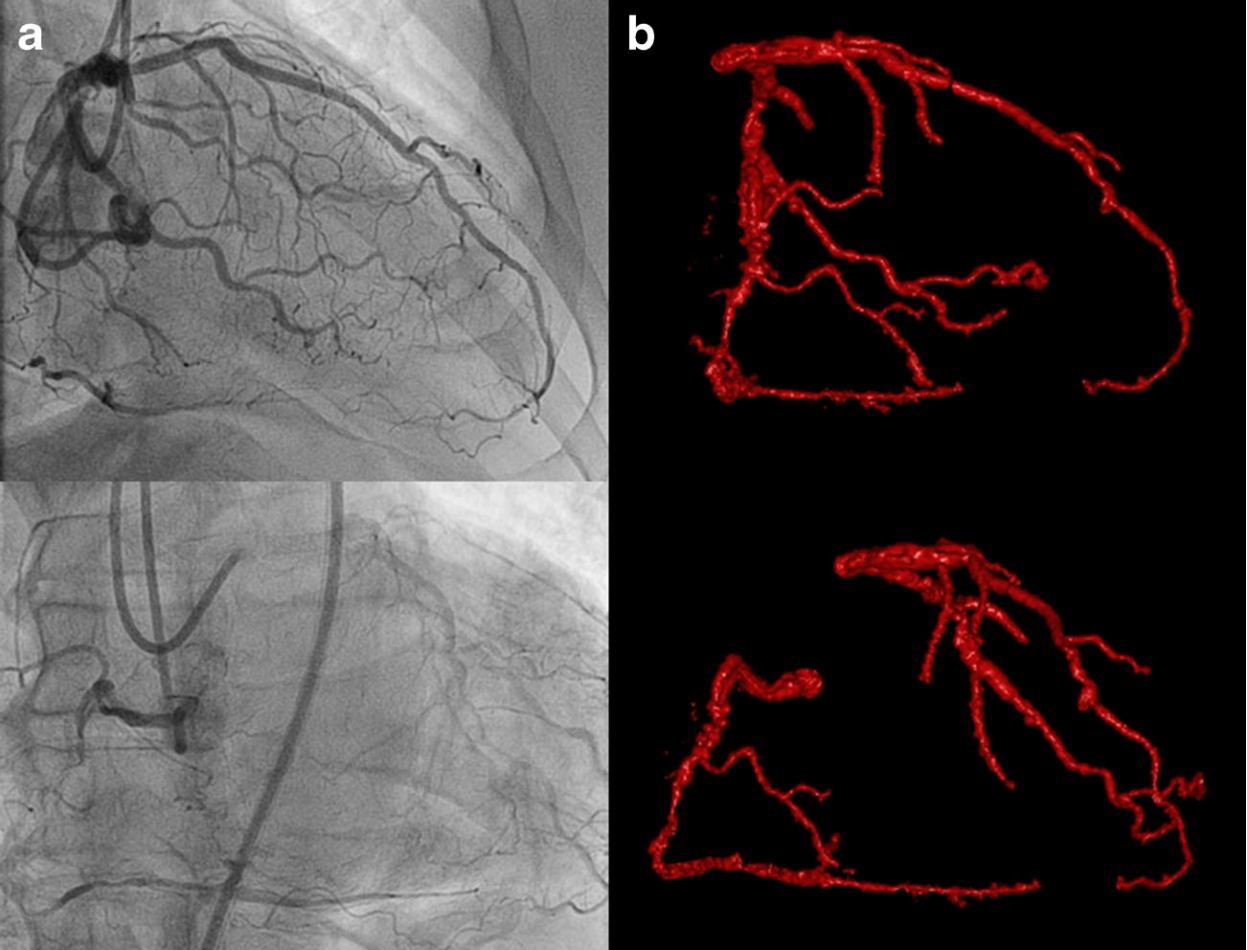
CCTA coronary course tracking. ICA of the RCA during bilateral contrast injection (**a**) and corresponding CCTA coronary course tracking images (**b**) show that the course of the occluded segment is superiorly illustrated with CCTA in comparison with ICA. Abbreviations as in Figs. 2, 3, and 5

**Fig. 10:**
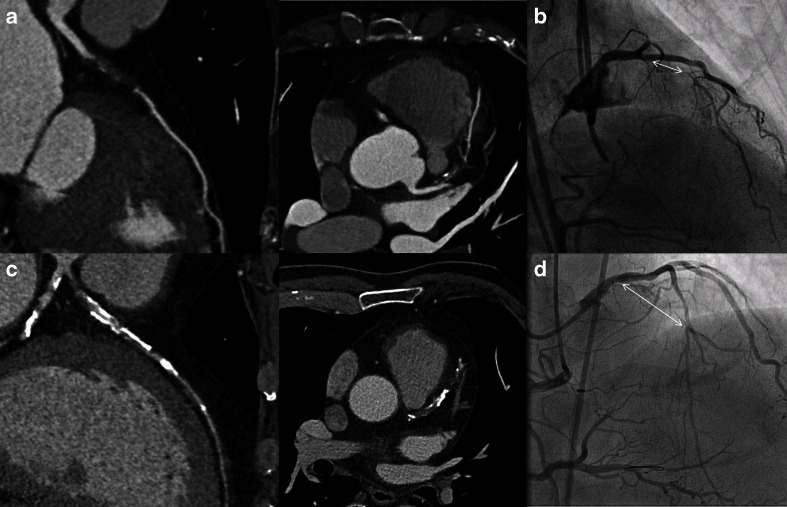
CTO wire crossing difficulty score appreciated from CCTA and ICA. The *top* CCTA (**a**) and ICA (**b**) images display a short occlusion (<20 mm) (*white arrow*), tapered proximal stump, no calcification, and no bending of the occluded segment (low CT-RECTOR score). This occlusion should be approached and probably successfully crossed with antegrade wire escalation technique. The *bottom* CCTA (**c**) and ICA (**d**) images clearly demonstrates a long (>20 mm) (*white arrow*) calcified occlusion (high CT-RECTOR score) suggesting that the operator should be able to use dissection and re-entry techniques before trying to cross this occlusion because anatomy dictates which approach should be used. Abbreviations as in Figs. 2 and 3

**Fig. 11:**
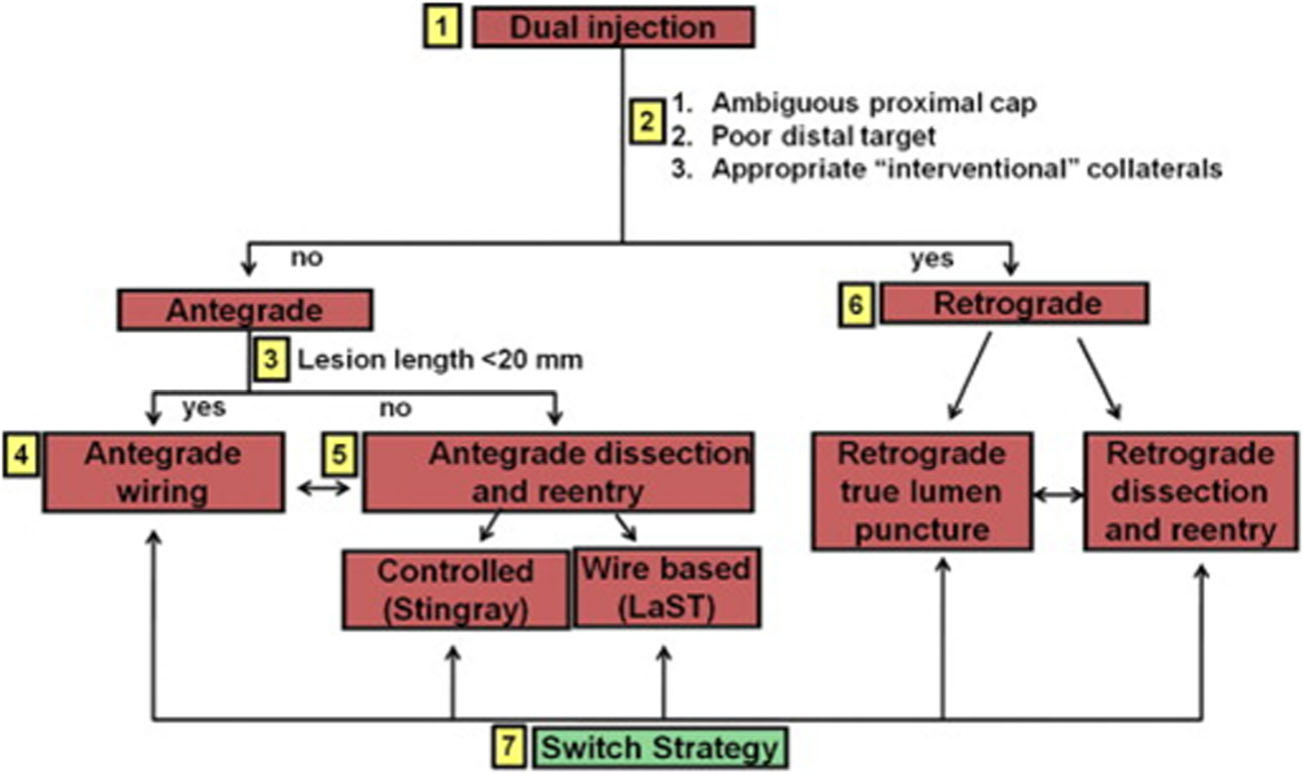
Algorithm for crossing CTOs. Hybrid approach dictated by anatomical features of a chronic total occlusion of a native coronary artery. *LaST* limited antegrade subintimal tracking; other abbreviations as in Fig. 2; reprint with permission [29••]

Historically, the success rate of PCI CTO ranged between 50 and 70 %, which has resulted in the reluctance to attempt percutaneous revascularization [1, 7, 12]. To more adequately predict the chances of successful antegrade wire crossing (within 30 min) for appropriate case selection, the Japanese Multicenter CTO Registry (J-CTO) score was developed [28•]. The J-CTO score has identified several independent angiographic predictors of failure being heavy calcification, bending within the occluded segment, blunt proximal stump, and occlusion length >20 mm. An additional point in this scoring system is given for a previous failed attempt. Low J-CTO scores are thus easily crossed, while high scores are characterized by high likelihood of failure. One of the issues in the full assessment of the J-CTO score is the frequent necessity of simultaneous double arterial injection of the right and left coronary arteries (see Figs. 2, 7, 8, and 9). These images are generally not produced during a diagnostic invasive angiogram. These angiographic features, however, can also be visualized by CCTA and could therefore similarly aid in the prediction of procedural success (Table 2). Several small-scaled studies have indeed identified very comparable angiographic CTO characteristics obtained with CCTA that were associated with PCI CTO failure as those observed in the J-CTO registry [74–78]. It has even been suggested that pre-procedural knowledge of the three-dimensional anatomy of the occlusion may aid in antegrade wire escalation and crossing of the lesion and thus improve procedural outcome [79]. Figure 9 illustrates that CCTA can be quite helpful to appreciate the coronary course of an occluded segment. More recently, Opolski et al. [80••] reported a CCTA-based scoring system in 240 CTO patients to predict success of percutaneous antegrade wire crossing in the Computed Tomography Registry of Chronic Total Occlusion Revascularization (CT-RECTOR). Very much like the J-CTO, independent angiographic predictors were occlusion length >20 mm, multiple occlusions, blunt stump, bending, and severe calcification. Clinical predictors were previously failed attempt and CTO duration >12 months or unknown duration. The predictive value of the CT-RECTOR proved to be superior over the J-CTO score to predict antegrade wire crossing <30 min (AUC 0.83 vs. 0.71). Figure 10 shows an example of a low and high CTO difficulty score on CCTA and ICA. These data suggest that CCTA could be utilized in clinical practice for case selection and perhaps even for pre-procedural planning to improve success. There is, however, an important caveat in the interpretation of these data. All of these studies have merely investigated the likelihood of antegrade wire crossing. Even though this type of approach is still the mainstay in attempts to percutaneously tackle CTOs, its success rate is low (62 % in the CT-RECTOR Registry). Dedicated CTO centers that have adopted the “hybrid” approach (i.e., next to antegrade wire escalation implementing retrograde wire escalation and dissection re-entry techniques) nowadays achieve success rates over 90 % with very acceptable complication rates. This approach (as depicted in Fig. 11) lets coronary anatomy define the procedural strategy, but anatomical features and lesion complexity are not used to select cases [29••]. As such, CCTA is hardly utilized by hybrid operators. Nonetheless, pre-procedural CCTA could be useful for antegrade wire operators. Based on non-invasive CT imaging, lesion complexity and thus chances of success can be accurately assessed and this information employed to select cases or refer to expert centers [81].

**Table 2:** Assessable angiographic features of a CTO

	Invasive	CCTA
Lesion length	++	+++
Tortuosity	++	+++
Calcification	+	+++
Proximal cap morphology	++	+
Proximal side branch	+++	++
Bridging collaterals	+++	+
True lumen flow	++	+
Vessel course	+	+++
Landing zone	++	++
Plaque characteristics	+	+++
Interventional collaterals	++	+

*CTO* chronic total occlusion, *CCTA* coronary computed tomography angiography

## Conclusions

Percutaneous revascularization of CTOs is less frequently performed as non-occlusive CAD due to technical procedural challenges, lower likelihood of recanalization, and increased complication rate. PET/CT allows for non-invasive imaging of myocardial perfusion and metabolism to objectify the extent of ischemia and viability. The hybrid images of coronary anatomy with CT and functional consequences of the CTO with PET allows for appropriate selection of patients who are likely to benefit from PCI CTO and facilitate procedural planning.

## Abbreviations

CAD: Coronary artery disease
CTO: Chronic total coronary occlusion
CABG: Coronary artery bypass grafting surgery
OMT: Optimal medical treatment
PCI: Percutaneous coronary intervention
LV: Left ventricular
PET: Positron emission tomography
CCTA: Coronary computed tomography angiography
ICA: Invasive coronary angiography
MPI: Myocardial perfusion imaging
SPECT: Single photon emission computed tomography
CMR: Magnetic resonance imaging
MPR: Multiplanar reconstructions
MIP: Maximum intensity projections
^18^F-FDG: ^18^F-deoxyglucose
